# Clinical aspects of deprescribing process in affective disorders

**DOI:** 10.1192/j.eurpsy.2021.183

**Published:** 2021-08-13

**Authors:** F. Bouckaert

**Affiliations:** Department Of Geriatric Psychiatry, University Psychiatric Center KU Leuven & Laboratory for Translational Neuropsychiatry, Leuven, Belgium

**Keywords:** Affective disorders, Elderly

## Abstract

Although depression in the elderly is often underdiagnosed and undertreated, some data show that next to this potential underuse, antidepressant prescriptions may also be overused and prescribed inappropriately. These potentially overused and inappropriate prescriptions of antidepressants are often related to polypharmacy, comorbidity and increased mortality. Deprescribing is the planned and supervised process of reducing or stopping medications that may no longer be of benefit or may be causing harm. Clinically relevant aspects and considerations of this deprescribing process in elderly patients with affective disorders will be discussed. Woodford HJ, Fisher J. New horizons in deprescribing for older people. Age and Ageing 2019;48:768-775. Hiance-Delahaye A, et al. Potentially inappropriate prescription of antidepressants in old people: characteristics, associated factors, and impact on mortality. Int Psychogeriatr 2018 May;30(5):715-726. Bobo WV, et al. Frequency and predictors of the potential overprescribing of antidepressants in elderly residents of a geographically defined U.S. population. Pharmacol Res Perspect 2019;e00461.

**Disclosure:**

No significant relationships.

